# Editorial: Brain Insulin Resistance in Neurodevelopmental and Neurodegenerative Disorders: Mind the Gap!

**DOI:** 10.3389/fnins.2021.730378

**Published:** 2021-08-10

**Authors:** Mara Dierssen, Eugenio Barone

**Affiliations:** ^1^Centre for Genomic Regulation (CRG), The Barcelona Institute of Science and Technology, Barcelona, Spain; ^2^Department of Experimental Sciences, Universitat Pompeu Fabra (UPF), Barcelona, Spain; ^3^Human Pharmacology and Clinical Neurosciences Research Group, Neurosciences Research Program, Hospital Del Mar Medical Research Institute (IMIM), Barcelona, Spain; ^4^Centro de Investigación Biomédica en Red de Enfermedades Raras (CIBERER), Valencia, Spain; ^5^Department of Biochemical Sciences “A. Rossi-Fanelli”, Sapienza University of Rome, Rome, Italy

**Keywords:** insulin resistance, neurodevelopment and brain metabolism, neurodegenerative diseases, learning and memory, brain insulin resistance, Alzheimer's disease, Down syndrome, Parkinson's disease

The failure of insulin signaling—a condition known as insulin resistance—is a key pathological feature of both type 2 diabetes (T2DM, systemic insulin resistance) (Arnold et al., 2018; Kellar and Craft, 2020) and brain disorders, such as Alzheimer disease and related dementias (ADRDs, brain insulin resistance) but also other neurodegenerative disorders such as Parkinson's or Huntington's disease (Brás et al., 2019; Hong et al., 2020), neurodevelopmental disorders such as Down syndrome (DS) (Tramutola et al., 2020; Lanzillotta et al., 2021) or autism (Manco et al., 2021) and behavioral disorders (Kleinridders et al., 2015), and greatly contributes to their pathogenesis (Arnold et al., 2018; Kellar and Craft, 2020). Specifically, regarding ADRDs and T2DM, considerable overlap has been found in the risk factors, comorbidities and putative pathophysiological mechanisms, leading to the proposal that AD is type 3 diabetes (Butterfield et al., 2014; de la Monte, 2019). Examination of postmortem AD, amnestic mild cognitive impairment and DS brains, uncovered key signs of brain insulin resistance, i.e., reduced insulin receptor (IR) and increased serine phosphorylation (inhibitory) of insulin receptor substrate 1 (IRS1), as well as reduced activation of pathways downstream from IRS1, particularly in the hippocampus, cortex, and hypothalamus (Talbot et al., 2012; Tramutola et al., 2015, 2020; Sharma et al., 2019). Higher levels of insulin resistance markers are associated with poorer performance on cognitive tests of episodic and working memory, independent of the load of senile plaques and tangles, thus suggesting a role for insulin signaling in neuronal functions (Talbot et al., 2012). At the cellular level, these dysfunctions might manifest as the impairment of neuroplasticity, receptor regulation or neurotransmitter release in neurons (Spinelli et al., 2017; Barone et al., 2019; Franklin et al., 2019; Melo et al., 2020; Lanzillotta et al., 2021), or the impairment of processes more directly implicated in insulin metabolism, such as neuronal glucose uptake in neurons expressing GLUT4, or homeostatic or inflammatory responses to insulin (Bomfim et al., 2012; Lourenco et al., 2013; Barone et al., 2016; Triani et al., 2018; Melo et al., 2020; Lanzillotta et al., 2021). Further, intense research over the last two decades has highlighted the impact of insulin signaling, brain energy balance, and their fluctuations on neurogenesis processes both during post-natal and adult life (Barone et al., 2014; Arnold et al., 2018; Kellar and Craft, 2020). Indeed, diabetes, obesity, and overweight are prevalent pregnancy complications that predispose offspring to neurodevelopmental disorders (Fusco et al., 2019; Dearden et al., 2020). Moreover, impaired neurogenesis compromises hippocampal function and plays a role in cognitive deficits in ADDRs (Arnold et al., 2018). In those plastic neural tissues, activation of insulin signaling regulates birth, specification, migration, and integration of newly generated neurons, suggesting that alterations of this key signaling transduction pathway may have a role both in neurodevelopmental disorders and adult neurogenesis (Arnold et al., 2018; Kellar and Craft, 2020).

The aim of this Research Topic is to outline the major needs and challenges in the comprehension of the role of insulin signaling and its alterations in the brain and how such alterations contribute to the development of neurodevelopmental and neurodegenerative disorders.

Recently, several studies evaluated the effects of nutrient-related signals on both brain development and cognitive functions. A key finding was the discovery that, other than hypothalamus, a number of brain regions express receptors for hormones known to regulate metabolic processes. In particular, insulin signaling was found to impact on molecular pathways underlying hippocampal plasticity, learning, and memory. Spinelli et al. discuss evidence linking the altered insulin sensitivity in the hippocampus with defects of both adult neurogenesis and synaptic plasticity. These authors also review epidemiological studies and the observations collected in experimental models focusing the attention on the critical role of brain insulin resistance at the crossroad between metabolic and neurodegenerative diseases.

One of the key questions regarding the effects of insulin within the brain, has to do with insulin uptake. Indeed, two sources for insulin in the brain have been identified: (1) circulating insulin produced by the pancreas enters into the brain through a receptor-mediated uptake at the level of blood brain barrier (BBB) (Rhea and Banks; Rhea et al., 2020); or (2) *in situ* synthesis by specific neuronal populations (Nakabeppu, 2019). While the second hypothesis is still under debate, the first one is the most accepted and validated by several studies. Rhea and Banks provide a comprehensive review about the role of the different cell types found at the BBB in regulating insulin uptake. Furthermore, they discuss how alterations of BBB favor the development of brain insulin resistance by stressing the role for the IR at the BBB, that goes beyond its canonical role in mediating the activation of the signal. Indeed, IR at the BBB is the main transporter for insulin within the brain. In this context, the review also addresses the effects of intranasal insulin administration and weight loss-associated improved insulin sensitivity, that are two validated strategies to increase brain insulin uptake and promoting neuroprotective effects.

From a molecular point of view, the activation of the insulin signaling requires IR-mediated tyrosine (Tyr) phosphorylation of IRS1. Following IRS1 activation, two main signaling cascades are activated downstream from IRS1: the mitogen-activated protein kinase (MAPK), and the phosphatidyl-inositol 3-kinase (PI3K)/protein kinase B (Akt). In the brain, MAPK are involved in the regulation of genes controlling synapse growth, neuronal maintenance, and repair processes while the PI3K/Akt pathway is involved in the maintenance of synaptic plasticity, stress response and neuronal metabolism and autophagy (Tramutola et al., 2016; Arnold et al., 2018). Gabbouji et al. highlight the role for the PI3K/Akt axis in the brain, showing how similar alterations were observed both in T2DM and AD within this pathway. Such molecular alterations drive the impairment of glucose uptake and metabolism as well as an increase of inflammatory processes within the brain and likely suggest that PI3K/Akt axis impairment could be a common denominator in those diseases.

Interestingly, the alteration of the PI3K/Akt axis in AD may result from accumulation of cholesterol oxidation products in the brain called oxysterols. Some oxysterols (e.g., 27-OHC, 7β-OHC, and 7-KC) deriving from cholesterol enzymatic oxidation or auto-oxidation further exacerbate cell-damage by sustaining free-radical chain reactions (Palozza et al., 2008; Niki, 2018). Furthermore, increased oxysterols levels were detected in AD brains compared to controls (Hascalovici et al., 2009; Testa et al., 2016). Gamba et al. address this topic by discussing how cholesterol oxidation products, i.e., oxysterols, may favor brain insulin resistance development, thus contributing to disrupt glucose uptake resulting in increased accumulation and reduced clearance of both Aβ and phospho-Tau in AD brain.

Similarly, Chatterjee et al. provide experimental evidence that the impairment of the PI3K/Akt axis due to insulin resistance favors Tau phosphorylation through a mechanism involving GSK3β and reduced autophagy in Drosophila. These authors show that co-expression of Chico (homolog of the mammalian IRS) and Tau leads to GSK3β inactivation and reduces Tau hyperphosphorylation in Drosophila. Conversely, the co-expression of insulin-resistant Chico loss of function results in hyper-active GSK3β and Tau hyperphosphorylation, thus suggesting that IRS1 would play a pivotal role in controlling downstream kinases in AD.

The detrimental impact of metabolic disorders, e.g., T2DM, obesity and metabolic syndrome, on brain structure and function has been also addressed. A research paper authored by Kavanagh et al. provides evidence that T2DM in vervet monkeys produces alterations in brain metabolism that foster the amyloidogenic pathway similar to what is observed in pre-symptomatic AD. This study shows that during the progression from healthy to pre-diabetes to T2DM, the brain moves into a state of altered metabolism that is characterized by higher glucose and lower amino acids and acylcarnitines levels. Then, increased cerebral metabolism seems to drive Aβ production and accelerates Aβ aggregation, in T2D similar to AD. These results shed light on the mechanisms through which T2DM development could lead to AD-related pathology and cognitive decline.

Movassat et al. briefly examined the main mechanisms linking T2DM to AD and provide the first evidence that certain circulating AD biomarkers can be found in goto-kakizaki (GK) rats, a model of non-obesity induced diabetes, suggesting that GK rats may be a model to investigate common molecular mechanisms of both disorders. Furthermore, Duarte et al. tested the hypothesis that caffeine exposure ameliorates T2DM-induced hippocampal alterations in GK rat brain. Caffeine is a non-selective antagonist of adenosine receptors (both A_1_R and A_2A_R), whose activity impact on the molecular processes regulating cognitive and learning functions. A_1_R and A_2A_R were found to be altered in the brain of T2D animal models. Caffeine-mediated neuroprotective effects were likely promoted through the reduction of A_2A_Rs activity at the synaptic level as well as in glial cells. Indeed, caffeine long-term intake was associated with improved memory functions, reduced astrogliosis, and reduced hippocampal synaptic degeneration in GK rats. Although long-term intake of caffeine did not prevent T2D-induced metabolic alterations in the hippocampus, its neuroprotective effects may be of help to delay the progression of T2D-related neurodegeneration.

With regard to obesity, Lloret et al. discuss the increased risk to develop AD for overweight and obese individuals, describing the role of obesity-associated hyper-leptinemia in promoting brain insulin resistance and glutamate-induced excitotoxicity. Under physiological conditions, insulin secretion stimulates leptin synthesis and release by adipocytes thus favoring satiety, whereby, as in a vicious cycle, leptin reduces insulin secretion and enhances insulin sensitivity to promote glucose uptake and metabolism. Moreover, leptin was shown to have neuroprotective functions by favoring long-term potentiation (LTP) and boosting the activity of N-methyl-D-aspartate (NMDA) receptors at synaptic levels. Conversely, increased circulating leptin levels would lead to leptin resistance and consequently to insulin resistance found to be associated with LTP dysfunction, and NMDA excitotoxicity in obese individuals and AD subjects. For that reasons, obesity in middle-age could be considered as a risk factor to develop AD in the elderly.

Among the intracellular processes dependent on the rate of glucose uptake, that are altered under insulin resistance conditions, O-GlcNAcylation post-translational modifications emerged as a key process regulating protein functions (Moll et al., 2020; Zuliani et al., 2021). Ansari and Emerald discuss the reciprocal interaction between insulin signaling and O-GlcNAcylation process in the brain, highlighting that increased O-GlcNAcylation of active sites of proteins of insulin signaling may promote the development of brain insulin resistance. In turn, brain insulin resistance, by reducing glucose uptake, likely prevent the O-GlcNAcylation process, thus impairing the activity of many proteins. In particular, reduced O-GlcNAcylation of amyloid precursor protein (APP) and Tau may be responsible for increased Aβ production as well as Tau phosphorylation, both processes associated with the development of AD.

Another key aspect that links metabolic disorders and development of neurodegenerative diseases is the lipid dysmetabolism (Trostchansky, 2019; Falabella et al., 2021). Indeed, insulin signaling, beyond glucose metabolism, also regulates lipid metabolism while insulin resistance leads to dyslipidemia (Arnold et al., 2018). By discussing current evidence, Le Stunff et al. propose that an excess of toxic lipids generated in the liver can be a cause of neurodegeneration. In particular, dyslipidemia may lead to increased ceramide levels that, due to their hydrophobic nature, can cross the BBB, thus promoting an exaggerate production of pro-inflammatory cytokines within the brain fostering development of brain insulin resistance and cognitive decline.

The relevance for brain insulin resistance in development of Parkinson disease (PD) was also described. Fiory et al. highlight how peripheral alterations, such those occurring in T2DM, have detrimental effects on PD, by negatively affecting PD phenotype, accelerating its progression and worsening cognitive impairment. These authors provide an extensive analysis of the recent lines of evidence supporting this idea, by showing how insulin resistance both in peripheral tissues and in the brain worsens functions of dopaminergic neurons, favors alpha-synuclein aggregation, impairs mitochondrial functions and promotes a pro-inflammatory state, that are all features of PD. Moreover, they provide an update about the neuroprotective effects of antidiabetic drugs on PD onset and progression collected both in humans and animal models.

Among the strategies to overcome T2DM-associated metabolic dysfunctions and cognitive decline the role of glucagon-like peptide-1 (GLP1) is gaining much attention. Indeed, GLP1 and insulin pathway share several targets downstream from IRS1, including Akt and MAPK, thus meaning that activation of GLP1 cascade may be useful to overcome IRS1 inhibition and thus insulin resistance (Tramutola et al., 2017; Holscher, 2019). Grieco et al. address this aspect by emphasizing how the increased activation of GLP1 signaling pathway obtained through the administration of GLP1 receptor (GLP1R) agonists in experimental models of AD, PD and T2DM promotes neuroprotective effects. These latter include reduced Aβ and hyperphosphorylated Tau, reduced oxidative stress and inflammatory processes and improved functions of dopaminergic neurons. Overall, by reducing neurotoxic events, GLP1R agonists ameliorate synaptic plasticity thus exerting beneficial effects on cognitive functions.

Finally, Dierssen et al. provide a comprehensive analysis of the literature addressing the role of insulin signaling in DS. DS is the most frequent chromosomal abnormality responsible for intellectual disability, due to the presence of an extra complete or segment of chromosome 21 (Hsa21). Furthermore, DS individuals are at high risk to develop AD after the age of 40. Multiple genes and factors are responsible for the major DS phenotypes and as explained in the review, insulin signaling in the brain is thought to mediate brain dysfunction associated with intellectual disability and the development of AD in DS.

## Author Contributions

All authors listed have made a substantial, direct and intellectual contribution to the work, and approved it for publication.

## Conflict of Interest

The authors declare that the research was conducted in the absence of any commercial or financial relationships that could be construed as a potential conflict of interest.

## Publisher's Note

All claims expressed in this article are solely those of the authors and do not necessarily represent those of their affiliated organizations, or those of the publisher, the editors and the reviewers. Any product that may be evaluated in this article, or claim that may be made by its manufacturer, is not guaranteed or endorsed by the publisher.
